# Social Media Listening to Understand the Lived Experience of Individuals in Europe With Metastatic Breast Cancer: A Systematic Search and Content Analysis Study

**DOI:** 10.3389/fonc.2022.863641

**Published:** 2022-05-20

**Authors:** Manuelita Mazza, Maria Piperis, Sathyaraj Aasaithambi, Jyoti Chauhan, Alexandros Sagkriotis, Claudia Vieira

**Affiliations:** ^1^ Divison of Medical Senology, European Institute of Oncology, IRCCS, Milan, Italy; ^2^ CyberKnife and TomoTherapy Department, Iatropolis Medical Group of Companies, Athens, Greece; ^3^ Insights and Analytics, Novartis Pharma AG, Basel, Switzerland; ^4^ Medical Oncology Department, Instituto Português de Oncologia do Porto Francisco Gentil (IPO-PORTO), Porto, Portugal

**Keywords:** secondary breast cancer, metastatic breast cancer, lived experience, social media listening, content analysis, sentiment analysis

## Abstract

**Background:**

Despite a wealth of real-world data on metastatic breast cancer (mBC), insights into the lived experience are lacking. This study aimed to explore how the lived experience of mBC is described on social media.

**Methods:**

A predefined search string identified posts relevant to the lived experience of mBC from Twitter, patient forums, and blogs across 14 European countries. The final data set was analyzed using content analysis.

**Results:**

A total of 76,456 conversations were identified between November 1, 2018, and November 30, 2020. Twitter was the most commonly used social media platform across all 76,456 conversations from the raw data set (*n* = 61,165; 80%). Automated and manual relevancy checks followed by a final random sampling filter identified 820 conversations for content analysis. The majority of data from the raw data set was generated from the United Kingdom (*n* = 31,346; 41%). From this final data set, 61% of posts were authored by patients, 15% by friends and/or family members of patients, and 14% by caregivers. A total of 686 conversations described the patient journey (*n* = 686/820; 84%); 64% of these (*n* = 439) concerned breast cancer treatment, with approximately 40% of discussions regarding diagnosis and tests (*n* = 274/686) and less than 20% of discussions surrounding disease management (*n* = 123/686; 18%). Key themes relating to a lack of effective treatment, prolonged survival and associated quality of life, debilitating consequences of side effects, and the social impacts of living with mBC were identified.

**Conclusions:**

The findings from this study provided an insight into the lived experience of mBC. While retrospective data collection inherently limits the amount of demographic or clinical information that can be obtained from the population sample, social media listening studies offer training to healthcare professionals in communication, the importance of quality of life, organization of healthcare, and even the design of clinical trials. As new targeted therapies are gradually incorporated into clinical practice, innovative technologies, such as social media listening, have the potential to support regulatory procedures and drug toxicity monitoring, as well as provide the patient voice in the regulation of new and existing medicines.

## Introduction

Breast cancer is the second most common cancer worldwide (1) and the most common among women, representing approximately one-quarter of all female cancer cases (2). Almost one-third of patients with a diagnosis of early-stage breast cancer subsequently develop metastatic breast cancer (mBC) (3). Although improvements in the detection and treatment of breast cancer have contributed to increasing survival rates in both non-metastatic and metastatic diseases (2), mBC continues to have a poor prognosis.

Median survival in patients with mBC is 2–4 years (4, 5) with a 5-year survival of less than 25% (6, 7). Treatment of mBC, therefore, involves palliative care, focusing on reducing disease burden, maintaining or increasing health-related quality of life, and prolonging life expectancy (8–10).

Despite a wealth of real-world data in mBC, insights into the lived experience using large data sets obtained *via* social media listening techniques are lacking. Traditional methods of gathering qualitative data in clinical research include questionnaires, surveys, interviews, and focus groups, as well as the collection of patient-reported outcome measures (11, 12). Typically, such studies involve relatively small numbers of participants (13). Previous qualitative research into the lived experience of mBC has highlighted a lack of information regarding treatment options and symptom management, as well as the physical and psychosocial impact of mBC on quality of life (14, 15). However, the rise of social media in recent years as an integral tool for communication and information gathering has transformed how patients learn about their condition, form peer support networks, and share their experiences (16).

While social media usage is typically highest among individuals 18–29 years of age (17), internet usage in general has increased in older populations in recent years (18), and social media usage among individuals 65 years of age and older has tripled in the past decade (17). Therefore, social media listening is emerging as a valuable technique for systematically reviewing the rich data that social media platforms offer across the age spectrum.

Previous social media listening studies have highlighted how Twitter has been used to share personal experiences, gather information, and obtain psychological support (19–21). More recently, targeted social media listening studies of chronic obstructive pulmonary disease and presbyopia have provided ecological validity to qualitative literature findings (22, 23). Although social media research focusing on the burden of illness in patients with mBC has previously been conducted (24), the authors are unaware of any previous social media listening study investigating the lived experience of patients with breast cancer.

The purpose of this study was to explore how the breast cancer community describes the lived experience of mBC on social media platforms, in order to provide perspectives and insights into living with or caring for someone with breast cancer. A secondary objective was to complement the qualitative literature on the lived experience of breast cancer.

## Methods

### Study Design

This was a non-interventional retrospective analysis of social media data available in the public domain, as opposed to a traditional systematic literature review.

### Search Strategy

A predefined search string was used to identify social media posts between November 1, 2018, and November 30, 2020, relevant to the lived experience of mBC from patient sources such as Twitter, patient forums, and blogs. The search string terms were built in each language to identify mBC-related posts/conversations, and Boolean operators (AND, OR) were used to combine individual keywords within the search strings. A preliminary search strategy allowed for appropriate terms to be included in the search strategy.

The search string (**Supplementary Appendix 1**) contained terminologies related to the following predefined research domains: epidemiology and patient characteristics, treatment patterns, burden of illness, real-world safety, real-world effectiveness, and predictors of outcomes. Generic search terms including “metastatic”, “advanced stage”, or “spreading cancer” were translated into 10 additional languages (Spanish, French, German, Dutch, Italian, Portuguese, Swedish, Norwegian, Danish, and Finnish) and included in the search strategy. Boolean operators (AND, OR) were used to define a single search string.

### Data Collection

Talkwalker (Talkwalker Sarl, Kirchberg, Luxembourg), a cloud-based social media aggregator tool, was used to conduct the predefined searches. The platform allows the analysis of millions of conversations from social media, news sites, blogs, and forums, allowing rich and relevant data to be collected and analyzed. Although Instagram was part of the aggregating source, this platform does not allow geographic tagging of data, and initial searches revealed that the relevancy of posts was minimal. Instagram was therefore filtered out from the data collection process.

During the data gathering process, data from openly available patient sources were retained, and data from non-patient websites (online pharmacies, pet/animal-related sites) were discarded. Information from closed platforms was unreachable and could not therefore be included.

The Talkwalker software identified social media posts from Austria, Belgium, Denmark, Finland, France, Germany, Italy, the Netherlands, Norway, Portugal, Spain, Sweden, Switzerland, and the United Kingdom. Data in native languages were then translated into English with the help of local translation experts.

### Data Analysis

The raw data set contained 2-year historic data (November 1, 2018, to November 30, 2020), which was processed to remove irrelevant content through keyword-based automated checks and an additional layer of human intelligence to remove any duplicates or irrelevant data. Data were subsequently contextualized to identify those conversations that answered at least one research question prior to content analysis (SA and JC). This second layer of filtering was conducted to remove any non-insightful data. Contextualized data were used to derive further samples for scoped research questions and deep dive.

**Figure 1:**
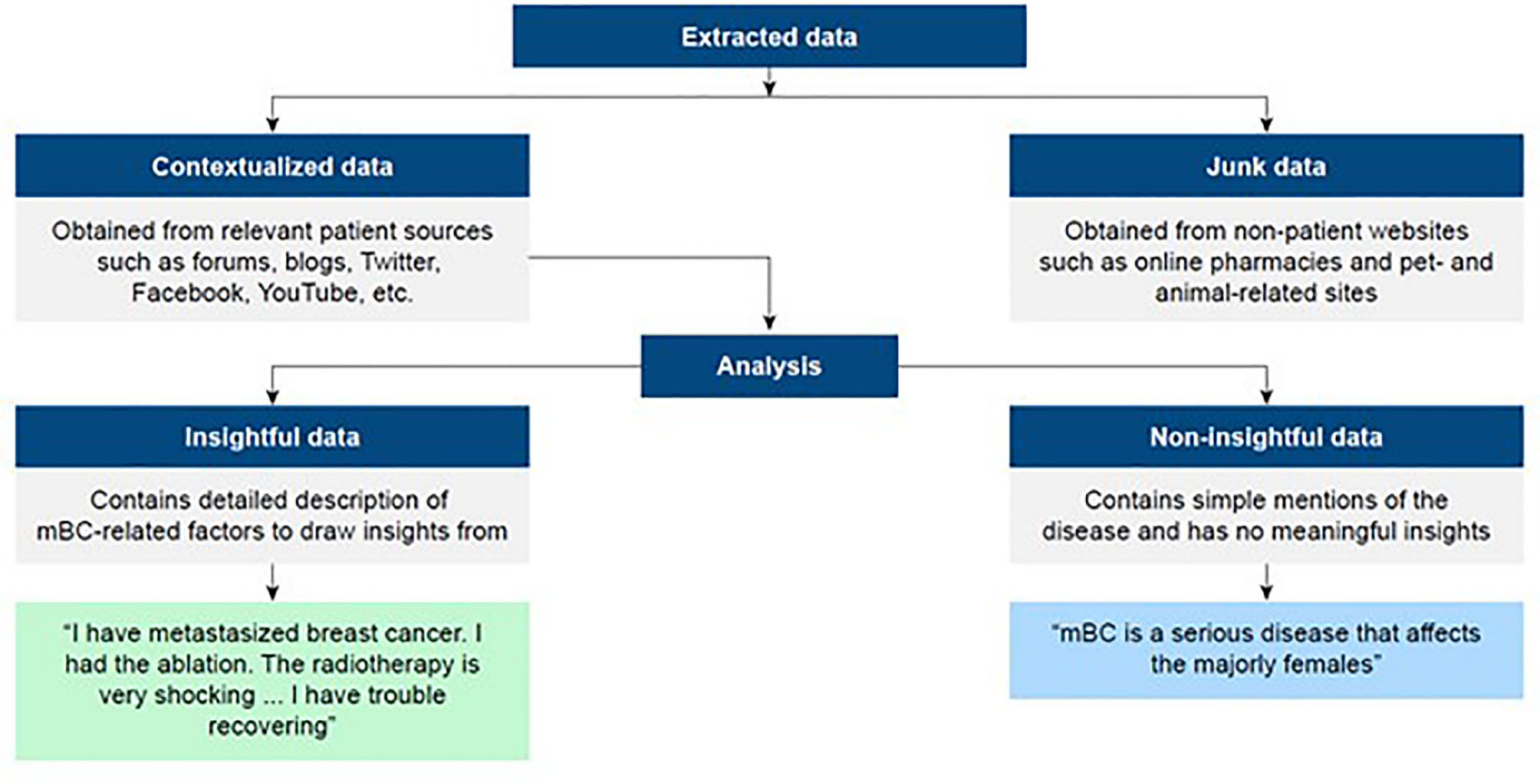
Data filtering process. mBC, metastatic breast cancer.

Taxonomies were developed to define insightful data as those that contained detailed descriptions of mBC-related factors and non-insightful data as those that contained a simple reference to the disease with no meaningful insight (**Figure 1**). Any images and videos were reviewed and insights extracted. Such instances were minimal, and a preference for conversing in the text was seen. An iterative random sampling procedure was applied to the sample per agreed proportions of social media records by country. **Figure 1** provides a further overview of the data collection and analysis process.

Talkwalker natural language processing was also used to perform sentiment analysis, categorizing discussion points into positive, negative, and neutral content.

## Results

### Epidemiology and Patient Characteristics

**Figure 2:**
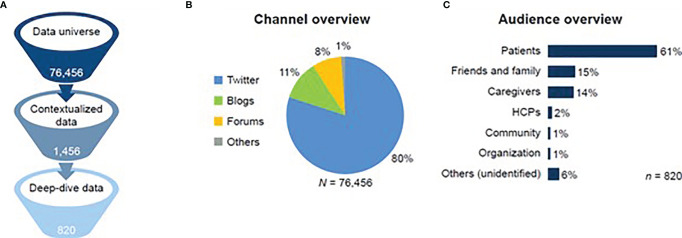
Flow diagram of social media post-selection and demographics of channel and audiences. **(A)** Data filtering stages. **(B)** Share of data universe by social media channel. **(C)** Share of deep-dive data by audience type. HCP, healthcare professional.

Social media posts were collated from November 1, 2018, to November 30, 2020, across selected European countries. A total of 76,456 conversations were identified in the raw data set (**Figure 2**). The relevancy check reduced this data set to 1,456 conversations, and the final filter identified 820 conversations.

The majority of data from the raw data set (*n* = 76,456) was generated from the United Kingdom (*n* = 31,346; 41%), Spain (12,998; 17%), and France (*n* = 8,410; 11%), with not more than 10% of conversations obtained from Germany (*n* = 7,646; 10%), Italy (*n* = 6,881; 9%), and the remaining European countries (1–4%) (**Table 1**).

**Table 1:** Origin of conversations by region.

Country/region	Share of total posts (%) *N* = 333	Share of country/region’s posts by channel (%)		
		Twitter	Blogs	Forum
United Kingdom	*n* = 137 (41%)	92%	7%	1%
Spain	*n* = 57 (17%)	96%	4%	0%
France	*n* = 37 (11%)	81%	13%	5%
Germany	*n* = 33 (10%)	25%	15%	59%
Italy	*n* = 30 (9%)	62%	32%	5%
Netherlands	*n* = 13 (4%)	76%	17%	6%
The Nordics*	*n* = 10 (3%)	60%	12%	28%
Belgium	*n* = 7 (2%)	85%	4%	11%
Switzerland	*n* = 3 (1%)	89%	8%	3%
Portugal	*n* = 3 (1%)	77%	23%	0%
Austria	*n* = 3 (1%)	49%	42%	10%

^*^The Nordics comprise Denmark, Finland, Iceland, Norway, and Sweden.

Twitter was the most commonly used social media platform across all 76,456 conversations from the raw data set (*n* = 61,165; 80%), apart from in Germany, where the primary channel for relevant posts was patient forums (*n* = 4,511; 59%). Blogs accounted for 11% (*n* = 8,410) of all 76,456 conversations, and forums accounted for 8% (*n* = 6,116) (**Figure 2**). Twitter and blogs were used primarily for raising awareness, while forums were typically used to seek or offer peer support. In-depth discussions about treatment typically took place on forums and blogs. Volume trend analysis demonstrated an increase in the volume of mBC-related conversations during notable holidays or health campaigns. Mentions increased during Breast Cancer Awareness month (October 2019 and October 2020) and in February 2020 when actress Shannen Doherty received a diagnosis of stage 4 breast cancer. During December 2019, a volume increase was seen, and close to Christmas, conversations tended to focus on the impact of mBC on patients and their families.

A total of 500 posts from the final data set were authored by patients (*n* = 500/820; 61%), 15% by friends and/or family members of patients (*n* = 123/820), and 14% (*n* = 115/820) by caregivers (**Figure 2**). Of those stakeholders whose gender was identified, 99% (*n* = 769/777) were female and 1% were male (*n* = 8/777). This finding was anticipated owing to the greater incidence of mBC among women. More men were identified in the United Kingdom than in other European countries.

The final data set captured 232 conversations in which the age of stakeholders was identifiable. The highest number of conversations was identified in social media users 31–40 years of age (*n* = 86; 37%), which is in line with social media use statistics. Age was more frequently identified in the United Kingdom and France than in other European countries. Greater than 50% of conversations (*n* = 137; 59%) were authored by stakeholders older than 41 years, which correlates with mBC statistics.

Conversations relating to late-stage breast cancer (defined by terms such as “metastatic”, “advanced stage”, or “spreading cancer”) accounted for 93% of posts in the final data set (*n* = 763/820). The majority of data collected did not further define the cancer type. However, 3% (*n* = 25/820) of conversations referred to a triple-negative diagnosis (mostly from Italy, France, the United Kingdom, and Spain), and 2% (*n* = 16/820) of conversations contained discussions surrounding HER2+ breast cancer (majority from France and the United Kingdom).

The region of metastasis was identifiable in fewer than 50% of conversations (*n* = 320/820; 39%). From the available data, the most common metastasized regions identified were bone (*n* = 128/320; 40%), liver (*n* = 112/320; 35%), and lung (*n* = 74/320; 23%).

In Germany, 99% of the relevant analyzed conversations were from forums, in which patients and caregivers shared their journey in search of advice and encouragement, often displaying a high level of knowledge about different drugs and diagnostic tools. Some German patients reported a lack of empathy and subsequent lack of trust in healthcare providers. **Table 2** highlights key country-specific metrics.

**Table 2:** Country-specific metrics.

Countries	Key patient journey stage	Key treatment types	Line of treatment	Treatment discontinuation	Key unmet needs
**UK 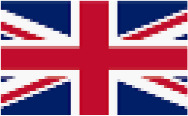 **	Treatment (56%) Diagnosis and tests (42%) Death (18%)	Chemotherapy (20%) Surgery (16%) Radiation therapy (15%)	1st line—100% (chemotherapy, targeted, surgery) 2nd line—53% (radiation)	Generic mentions of treatment discontinuation Due to COVID-19	Availability of effective treatment Lack of public awareness on how to offer support/care Safe access to care during COVID-19 Lack of effective screening Access to good HCPs or treatment
	*n* = 154	*n* = 87	*n* = 15		*n* = 89
**Spain 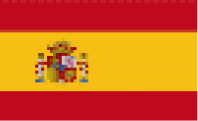 **	Treatment (49%) Diagnosis and tests (30%) Death (16%)	Chemotherapy (25%) Alternative (11%) Surgery (9%) Radiation therapy (9%)	1st line—100% (chemotherapy, hormonal, surgery) 2nd line—38% (radiation, surgery)	Side effects Inefficacy	Need for more research in the area for better treatment options Safe access to care during COVID-19 Lack of trust in HCP
	*n* = 89	*n* = 44	*n* = 24		*n* = 46
**France 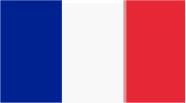 **	Treatment (70%) Diagnosis and tests (27%) Recurrence (26%)	Chemotherapy (32%) Surgery (28%) Hormonal therapy (25%)	1st line—68% (chemotherapy, hormonal, adj. chemo., surgery, targeted) 2nd line—77% (hormonal, chemotherapy, surgery, adj. chemo., immunotherapy)	Disease progression Doctor’s advice Side effects Inefficacy	Availability of effective treatment Lack of effective screening Lack of empathy from HCP
	*n* = 103	*n* = 72	*n* = 22		*n* = 8
**Italy 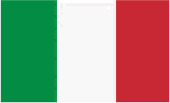 **	Treatment (57%) Management (38%) Diagnosis and tests (36%)	Hormonal therapy (30%) Targeted therapy (27%) Chemotherapy (27%)	1st line—100% (chemotherapy, surgery, targeted, immunotherapy) 2nd line—47% (chemotherapy, hormonal)	Side effects Inefficacy Disease progression Disease cured Doctor’s advice	Availability of effective treatment Delays in treatment/HCPs act too slowly Lack of communication from HCP
	*n* = 95	*n* = 64	*n* = 17		*n* = 11
**Germany 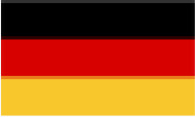 **	Treatment (79%) Diagnosis and tests (50%) Symptoms (25%)	Chemotherapy (29%) Targeted therapy (25%) Hormonal therapy (19%)	1st line—100% (chemotherapy, radiation, surgery) 2nd line—81% (chemotherapy, surgery)	Side effects Inefficacy Disease progression Doctor’s advice	Treatment side effects Lack of trust in HCP Lack of empathy and support from HCP Availability of an effective treatment
	*n* = 136	*n* = 107	*n* = 16		*n* = 33
**Netherlands 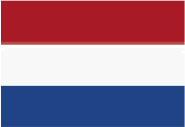 **	Treatment (81%) Diagnosis and tests (37%) Death (21%)	Chemotherapy (29%) Surgery (20%) Hormonal therapy (20%)	1st line—91% (chemotherapy, surgery) 2nd line—45% (surgery, hormonal)	Disease progression Doctor’s advice Inefficacy	Lack of public awareness on how to offer support/care Impossible to visit due to COVID-19 Lack of communication by HCP Lack of trust in HCP
	*n* = 43	*n* = 35	*n* = 11		*n* = 13
**The Nordics 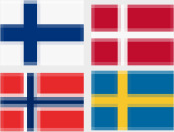 **	Treatment (69%) Diagnosis and tests (59%) Symptoms (41%)	Adj. chemotherapy (25%) Surgery (20%) Chemotherapy (20%)	1st line—100% (surgery, chemotherapy) 2nd line—43% (chemotherapy)	Doctor’s advice Disease cured	Treatment side effects Insurance/financial Access to good HCPs or treatment Availability of an effective treatment
	*n* = 29	*n* = 20	*n* = 7		*n* = 10
**Switzerland 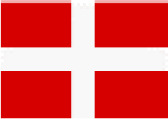 **	Treatment (100%) Symptoms (50%)	Palliative care (50%) Generic mentions of medicine (50%)	NA	NA	NA
	*n* = 2	*n* = 2			
**Austria 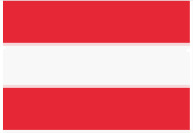 **	Diagnosis and tests (79%) Treatment (63%) Symptoms (47%)	Radiation therapy (64%) Targeted immunotherapy (45%) Hormonal, chemotherapy (27% each)	1st line—100% (chemotherapy) 2nd line—100% (radiation)	Generic mentions of discontinuation	Treatment side effects
	*n* = 19	*n* = 11	*n* = 3		*n* = 4
**Belgium 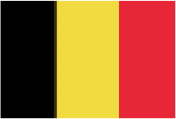 **	Death (43%) Diagnosis and tests (36%) Treatment (21%)	Generic mentions of “treatment” (20%)	NA	NA	Availability of an effective treatment Lack of awareness on providing support/care Lack of trust in HCPs
	*n* = 14	*n* = 2			*n* = 3
**Portugal 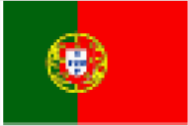 **	Diagnosis and tests (50%) Causes (50%)	NA	NA	NA	Insurance/financial Lack of empathy from HCP
	*n* = 2				*n* = 2

HCP, healthcare professional; NA, not applicable.

### Patient Journey

A total of 686 conversations from the final data set described the patient journey (*n* = 686/820; 84%). Greater than 50% of these conversations (*n* = 439/686; 64%) concerned breast cancer treatment, with approximately 40% of discussions regarding diagnosis and tests (*n* = 274/686), and fewer than 20% of discussions surrounding disease management (*n* = 123/686; 18%).

Pre-diagnosis symptoms were identifiable in 41 out of 686 patient journey conversations. Within these discussions, pain (*n* = 19/41; 46%) and a lump, thickening, or swelling in the breast (*n* = 15/41; 37%) were the most frequently cited pre-diagnosis symptoms. Dismissal and misdiagnosis of these symptoms were apparent from stakeholders in the United Kingdom and France.

CT scan was the most often mentioned diagnostic test, particularly in Germany, but was also visible in the majority of the other markets. Biopsy and PET scans were equally visible in most markets. Mammography was most commonly discussed in the Nordics (Sweden, Norway, Denmark, and Finland), France, Spain, and the United Kingdom. Mentions of diagnostic tests on social media platforms included sharing positive and negative outcomes, such as clean scans, recurrence, or new metastasis.

Approximately 3% (*n* = 22/686) of conversations discussed the cause of cancer, including hereditary and genetic factors. Social media users who shared this information were largely from the United Kingdom, Germany, France, Italy, and the Netherlands. Genetic mutations mentioned included *HER2*, *PTEN*, *BRCA1*, and *PIK3CA*. Patients mentioning mutations were aware of the implications of mutations with respect to treatment options. However, no reason could be attached to the low discussion levels around genetic factors of mBC across social media platforms.

The most common symptoms that patients experienced throughout their cancer journey (i.e., during diagnosis, treatment, management, remission, and/or recurrence) were reflected in 11% of conversations (*n* = 75/686). These mentions referred to treatment side effects more often than symptoms of cancer itself. Pain due to metastases, as well as weakness and fatigue, was the most-discussed symptom that related directly to the disease.

### Treatment Patterns

The line of therapy was discussed in approximately 16% of posts (*n* = 115/820). Chemotherapy, surgery, and targeted therapy were the most commonly discussed first-line therapies, accounting for 93% of conversations (*n* = 107/115). A similar trend was noticed for second-line therapies, in which chemotherapy and surgery were followed by hormonal therapy (*n* = 66/115; 57%). Radiation and hormonal therapy were the most prominent third- and fourth-line treatments (*n* = 29/115; 25% and *n* = 10/115; 9%, respectively). A summary of references to treatment types is contained in **Table 3**.

**Table 3:** Prevalence of cancer treatment types.

EU total *n* = 444	*N*/*n*		Chemotherapy (26%)	Surgery (17%)	Hormonal (17%)	Targeted (14%)	Radiation (13%)	Adjunctive chemotherapy (10%)	Targeted immunotherapy (5%)	Immunotherapy/biological (5%)	Palliative care (2%)	Others (47%)
**UK**	87		20%	16%	5%	3%	15%	10%	7%	3%	2%	54%
**Spain**	44		25%	9%	5%	0%	9%	5%	0%	5%	2%	66%
**France**	72		32%	28%	25%	15%	18%	14%	1%	6%	3%	54%
**Italy**	64		27%	19%	30%	27%	3%	19%	2%	6%	0%	20%
**Germany**	107		29%	14%	19%	25%	12%	3%	9%	2%	0%	42%
**Netherlands**	35		29%	20%	20%	3%	17%	3%	3%	11%	14%	51%
**The Nordics**	20		20%	20%	15%	5%	5%	25%	0%	15%	0%	50%
**Switzerland**	2		0%	0%	0%	0%	0%	0%	0%	0%	50%	50%
**Austria**	11		27%	9%	27%	9%	64%	18%	45%	9%	0%	36%
**Belgium**	2		0%	0%	0%	0%	0%	0%	0%	0%	0%	100%
**Portugal**	0		NA	NA	NA	NA	NA	NA	NA	NA	NA	NA

**Table:** 

				*High to low prevalence*								Total % may be >100% due to mention of multiple themes in posts.

NA, not applicable.

#### Chemotherapy

Chemotherapy was the most-discussed treatment among 444 mentions (*n* = 115; 26%) and the most prominent first-line therapy among 107 mentions (*n* = 42; 39%). On analyzing adjuvant and neoadjuvant therapies, chemotherapy was primarily mentioned both before and after surgery (*n* = 22 mentions). Key treatment combinations before surgery included chemotherapy + surgery (*n* = 3/6; 50%), while chemotherapy + radiation was the most common post-surgical therapy discussed (10/16; 63%). Patients who mentioned duration of time on treatment (*n* = 133) typically referenced 1–2 or 5–10 years (both 22%). Chemotherapy was the most mentioned therapy within the 1–2 years’ bracket.

Sentiment analysis revealed positive mentions due to efficacy and negative mentions due to the heavy burden of side effects:

*“I finished my 6th cycle of oral #chemo drug #Capecitabine - it’s still working”*

*“… chemotherapy and radiotherapy which make us sick to death … vomiting, fatigue, but the lump is reduced to zero!!!”*

Of the chemotherapy discussions, 20% were positive (*n* = 23/116) and 27% were negative (*n* = 31/116). The remaining 53% of discussions (*n* = 62/116) were categorized as neutral (**Table 4**).

**Table 4:** Sentiment regarding treatment types.

Sentiment—treatment types			Total *N*/*n*	Positive (%)	Negative (%)	Neutral (%)
Chemotherapy			116	20%	27%	53%
Surgery			77	13%	9%	78%
Hormonal therapy			76	24%	14%	62%
Targeted therapy			61	34%	15%	51%
Radiation therapy			59	10%	17%	73%
Adjunctive chemotherapy			44	20%	16%	64%
Targeted immunotherapy			24	25%	13%	63%
Immunotherapy/biological			23	39%	4%	57%

**Table:** 

				*High to low prevalence.*

Although there were no specific reasons cited for preferring chemotherapy over other options, it was usually given as a standard treatment before surgery to shrink the tumor size and post-surgery to prevent metastasis. It was considered by those posting to be effective and easily covered by health insurance in comparison to other newer therapies. Reasons for shifting from chemotherapy to another treatment option were rarely mentioned.

#### Surgery

Surgery was the second most-discussed breast cancer treatment, accounting for 17% of social media mentions (*n* = 75/444). Sentiment analysis revealed that 13% of surgical discussions were positive (*n* =10/77):

*“My sister had advanced breast cancer at the age of 30 and fought for a year with a mastectomy, chemo and radiotherapy … The doctors in the U.K. are amazing.”*

Of the surgical discussions, 9% were negative (*n* = 7/77) and 78% were neutral (*n* = 60/77).

#### Hormonal and Targeted Therapy

Hormonal and targeted therapies were mentioned in 17% (*n* = 76/444) and 14% (*n* = 62/444) of discussions, respectively. Sentiment analysis revealed that targeted therapy had a higher rate of positive categorization than hormonal therapy [34% (*n* = 21/61) vs. 24% (*n* = 18/76)]. Negative connotations were similar across both therapies, accounting for approximately 15% of mentions (*n* = 9/61 and *n* = 11/76, respectively).

#### Radiation Therapy

Radiation was mostly mentioned as third-line therapy and accounted for 13% of conversations (*n* = 58/444). Positive sentiments were obtained from 10% of discussions (*n* = 6/59), predominantly from social media users in the United Kingdom. Side effects of treatment largely attributed to 17% of mentions being assigned as a negative sentiment (*n* = 10/59). These negative sentiments were most frequently seen in social media users located in France and Germany who described the side effects as being difficult to tolerate.

### Stakeholder Perceptions of Effectiveness

Efficacy, treatment duration, and treatment sequence were the most commonly discussed treatment topics based on 329 conversations across social media platforms (43%, 40%, and 35%, respectively) (**Table 5**). A total of 49 out of 329 conversations (15%) related to a lack of efficacy associated with chemotherapy, hormonal therapy, targeted therapy, or surgery. The majority of these mentions were from stakeholders in Germany, France, and Italy.

**Table 5:** Treatment feature topics of discussion.

EU total *n* = 329	Total (*N*/*n*)	Efficacy (43%)	Treatment duration (40%)	Treatment sequence (35%)	Side effects (18%)	Frequency and dosage (17%)	Inefficacy (15%)	Others (33%)
**UK**	58	38%	38%	26%	12%	14%	5%	48%
**Spain**	39	41%	46%	62%	13%	3%	8%	21%
**France**	56	64%	55%	39%	25%	21%	21%	16%
**Italy**	53	62%	40%	32%	2%	4%	19%	49%
**Germany**	64	28%	13%	25%	31%	31%	27%	13%
**Netherlands**	30	27%	53%	37%	13%	13%	10%	53%
**The Nordics**	17	35%	65%	41%	29%	29%	0%	71%
**Switzerland**	2	0%	100%	0%	0%	0%	0%	0%
**Austria**	7	29%	29%	43%	57%	43%	14%	29%
**Belgium**	3	0%	67%	0%	0%	0%	0%	33%
**Portugal**	0	NA	NA	NA	NA	NA	NA	NA

**Table:** 

				*High to low prevalence.*								NA, not applicable.

Prolonged survival was the most prevalent clinical endpoint mentioned across all countries (*n* = 93/164; 57%), compared with improved quality of life (*n* = 20/164; 12%), curing cancer (*n* = 16/164; 10%), and remission (*n* = 16/164; 10%) (**Table 6**). Among stakeholders, patients were the most likely to discuss clinical endpoints.

**Table 6:** Clinical endpoints discussed.

	Clinical endpoints	Total *N*/*n*	Survivability/prolong survival (%)	Improved quality of life (%)	Curing or getting rid of cancer (%)	In remission (%)	Morbidity/mortality (%)	Others (%)
**Countries**	EU	164	57	12	10	10	9	30
	UK	22	59	0	18	18	0	41
	Spain	17	47	18	12	18	0	6
	France	26	73	12	4	23	0	31
	Italy	37	81	24	16	5	0	27
	Germany	16	13	13	0	6	0	69
	Netherlands	25	40	8	0	4	40	28
	The Nordics	12	75	0	17	0	8	8
	Switzerland	1	100	0	0	0	0	0
	Austria	2	0	0	0	0	0	100
	Belgium	6	33	0	17	0	50	0
	Portugal	0	NA	NA	NA	NA	NA	NA

**Table:** 

				*High to low prevalence.*								NA, not applicable.

For many patients, it was clear that prolonged survival was the treatment driver with little expectation of a cure. In the United Kingdom, although social media users recognized the incurable nature of mBC and prolonged survival with treatment, it was often viewed as a “death sentence”.

Treatment duration was discussed in terms of prolonged survival, time spent fighting the disease, or the efficacy of treatment up to relapse or a new metastasis. Discussions surrounding survival are often related to milestones, such as living beyond a given prognosis. Patients expressed terms such as “still here” and “still fighting” when conveying their feelings in relation to such milestones.

Tones of resignation and guarded optimism were commonly associated with discussion of prolonged survival. Patients expressed gratitude that they might be able to spend more time with their loved ones but also feared that cancer would come back. Sentiments such as feeling tired but fighting for loved ones were often seen.

### Real-World Safety

Treatment being ineffective, side effects, disease progression, and doctor’s advice were mentioned equally as reasons for treatment discontinuation (*n* = 8/41; 20% per category). Several generic mentions of discontinuing treatment were also encountered, in which an explanation was not available.

Side effects were associated with all treatment types and discussed in 59 out of 329 conversations (18%). The most common side effects referenced included fatigue, aches, sleep disorders, and deterioration in blood levels. Struggling with side effects of medicines was reported in 35 out of 219 patients (16%). This was more visible in Germany, the United Kingdom, Austria, and the Nordics.

### Burden of Illness and Quality of Life

Overall, 41% of stakeholder conversations (*n* = 333/820) discussed factors associated with quality of life, including emotional (*n* = 200/333; 60%), physical (*n* = 147/333; 44%), social (*n* = 57/333; 17%), and financial factors (*n* = 30/333; 9%).

Emotional factors were strongly associated with social media users in most countries, with stakeholders feeling emotionally drained by their mBC. Many patients linked negative feelings such as shock, disbelief, and fear to the moment of diagnosis. Feelings of “despair” and “grief”, as well as anxiety surrounding the future or the future of loved ones, were also reported.

*“Today is my 13th wedding anniversary … I thought we’d be married for decades. Thanks to my #stage4 #BreastCancer I don’t know how much longer we have together. I’m just hoping that 13 doesn’t [turn] out to be unlucky.”*

The physical impact of mBC was mentioned by 147 out of 333 stakeholders (44%) and was largely associated with treatment side effects (*n* = 53/147; 36%), and weakness and fatigue were prominently mentioned in 28% of discussions (*n* = 41/147). Some patients were resigned to the inevitability of treatment side effects, while others expressed despair. Social media users in France were more likely to express a stoical attitude towards pain, stating that it was something that naturally accompanies cancer and thus needed to be accepted.

Vitamins and supplements were the alternative measures mentioned most often across countries. However, these were not taken to replace conventional therapies but to support the body, boost the immune system, and help the patient to minimize the symptoms of cancer or treatment side effects. Physical pain and pain management, including medication, cannabidiol, and music therapy, were described by 34 out of 147 mentions (23%).

The social impact of mBC included an inability to continue working or being treated differently in the workplace. Twenty-four out of 58 discussions (41%) surrounded the effect cancer had on work, while 36% of discussions (*n* = 21/58) highlighted the impact of cancer on a patient’s social life:

*“Since I got sick, metastatic breast cancer in the lungs, I am often not “seen” by people I know. They hesitantly wave a hand and disappear into a shop.”*

Many patients underlined the importance of having a normal and fulfilling life and discussed traveling, enjoying themselves, and spending time with friends and family. Social media users in the United Kingdom appeared to particularly struggle with social restrictions implemented during the COVID-19 pandemic:

*“I have stage IV breast cancer I’m shielding. My 80yr old parents are shielding. I have limited time on this earth, but I haven’t broken lockdown to see them.”*

The financial implications of mBC were cited in 30 out of 333 mentions (9%). Some users described exhausting all of their personal resources, while others were engaged in seeking funding for treatments that were not covered by their insurance. When stakeholders talked about their need for financial support, they referred not only to medical financial support but also to economic support needed to cover basic needs, because the disease left them unable to work and, thus, they ended up in a precarious financial situation:

*“I have stage4 breast cancer … my daughter was sacked because she needed to take care of me.”*

Treatments that required financial support were unspecified innovative treatments or immunotherapy, although a few cases were encountered in which patients could not afford the co-payment for chemotherapy.

### Emergence of Key Unmet Needs

The lack of effective treatments (*n* = 42/219; 19%) and management of side effects (*n* = 35/219; 16%) were the two key unmet needs revealed during content analysis. These themes were followed by a lack of awareness of providing support or care (*n* = 35/219; 16%) and safe access to care during the COVID-19 pandemic (*n* = 28/219; 13%) (**Table 7**).

**Table 7:** Key unmet needs.

Key unmet needs	EU	UK	Spain	France	Italy	Germany	Netherlands	The Nordics	Switzerland	Austria	Belgium	Portugal
**Total *N*/*n***	**219**	**89**	**46**	**8**	**11**	**33**	**13**	**10**	**0**	**4**	**3**	**2**
Availability of an effective treatment	19%											
Treatment side effects	16%											
Lack of awareness on providing support/care	16%											
Safe access to care during COVID-19	13%											
Need for more research in the area for better treatment options	10%											
Lack of effective screening	9%											
Access to good HCPs or treatment	8%											
Delays in treatment/HCPs act too slowly	7%											
Lack of empathy, support, and communications from HCP	7%											
Lack of trust in HCP	5%											
Insurance/financial	5%											
Others	12%											

**Table:** 

			*High to low prevalence*.									HCP, healthcare professional.

Lack of an effective treatment was the most visible unmet need in the United Kingdom, France, Germany, the Nordics, Italy, and Belgium:

*“Today I found out that my nana … had cancer return as stage 4 breast cancer that’s metastasized into her bones. There’s no cure. 5 year survival rate is 22%.”*

Treatment side effects were reported in the United Kingdom, Austria, and the Nordic countries and were the most-discussed unmet need in Germany accounting for half of all conversations addressing unmet needs (**Table 7**). Side effects were discussed by some in terms of being a “necessary evil”; others reported that they had led to the discontinuation or suspension of treatment.

Stakeholders in the United Kingdom, the Netherlands, Spain, Italy, and Belgium highlighted a lack of understanding of how to provide support and care. Some patients reported that people did not know how to approach them, causing social isolation. Others presented complaints about their healthcare professionals; this could be because they distrusted their opinion regarding treatment or diagnosis, or because they did not feel supported. A lack of empathy, poor communication, and lack of time for answering questions were all reported.

Safe access to care during COVID-19 was identified as a problem in the United Kingdom, Spain, Germany, the Nordic countries, and the Netherlands. In the Netherlands, the policy-related hashtag #dorhout (#DryWood) was adopted by younger social media users to raise injustice between age and COVID-19 policies. In the United Kingdom, discussions regarding inequalities between primary cancer and secondary cancer care were a key trend, equating treatment delays with a death sentence:

*“My sister in law had her chemotherapy cancelled before it started for advanced breast cancer. It’s a death sentence, these deaths should be included in the final death numbers caused by COVID19.”*

## Discussion

### Overview of Findings

Understanding the lived experience of a disease is an important part of clinical practice and medicine development. Social media listening studies are therefore important for the training of healthcare professionals in communication, for the organization of healthcare that is more focused on the patient and their quality of life, and even for the design of clinical trials with drugs or strategies that can incorporate the information collected by this means.

While there are clear advantages of traditional qualitative techniques, such as interviews, focus groups, or patient surveys, they may limit the scope of the data collected by focusing on specific questions. Furthermore, these manual techniques can be costly and labor-intensive to administer, hindering the collection of large-scale data sets. Innovative technologies and social networks, such as blogs, forums, Twitter, and Facebook, represent a new and growing repository of publicly available, user-generated data that offer candid, first-hand, real-world accounts of what it is like to live with a particular disease. Topics of discussion on such platforms range from treatment, quality of life, disease progression, to unmet needs (25, 26). Social media listening studies, therefore, represent a methodology for exploring these large, searchable, and freely available data repositories.

In this study, social media platforms were used for various purposes, ranging from advocacy and awareness (which tended to take place on Twitter) to more in-depth discussions of therapies and the patient journey (more commonly occurring on forums). Volume trend analysis demonstrated an increased volume of mBC-related conversations during notable holidays or health campaigns. In this study, a peak in social media discussions was observed in October 2019 and October 2020 during Breast Cancer Awareness month. These findings support those of Kaleem et al., who found that the search term “breast cancer” consistently had a peaked interest during October in a retrospective analysis of Google search trends between 2004 and 2017 (27). The hashtag #IAmThe31 and #BusyLivingWithMets were seen in relation to raising awareness of mBC, particularly among stakeholders in the United Kingdom. In this study, a volume trend was seen in February 2020 when actress Shannen Doherty announced that she was battling stage 4 breast cancer, supporting existing research into the impact of celebrities on public health awareness (28).

Approximately 3% of conversations (*n* = 25/820) discussed the cause of cancer, including hereditary and genetic factors. While no reason could be attributed to the low discussion levels on social media, those mentioning mutations were aware of the implications with respect to treatment options. The impact of celebrities on public health awareness has also been shown to significantly increase genetic testing and mastectomy rates (28–30). The findings in this study further highlight the need for clinicians to be mindful of the impact celebrities and the media have on the delivery and management of cancer-related services.

Social media also appears to be a particularly beneficial forum for exploring the emotional and psychological impact of living with mBC, with emotional matters being discussed much more commonly than the social or financial consequences of cancer. Stakeholders engaged in candid discussions about their feelings, speaking of the shock of diagnosis and a sense of grief and anxiety when thinking about the future. Stakeholders also used social media to articulate the psychosocial impact of COVID-19 on mBC-related care. They expressed frustration about treatment delays and discontinuation, as well as social restrictions because a shift in healthcare priorities moved towards COVID-19, which had a dramatic effect on healthcare policies and practices across Europe in the latter quarter of the analysis period. These findings correlate with a recent evaluation of the impact of COVID-19 on the organization of oncological care in 18 European countries; cancellation or delay of surgery and chemotherapy were reported as the most affected treatment modalities (44% and 26%, respectively) (26). Earlier cessation of palliative treatment was observed in 32% of centers surveyed, and more than half of participating oncologists agreed that undertreatment was a major concern (*n* = 70/109; 64%) (31).

Key themes to emerge from this study were as follows: the lack of effective treatment, the importance of prolonged survival and quality of life, the debilitating consequences of side effects, and the social impacts of living with breast cancer. These findings were not surprising. mBC continues to have a poor prognosis, with a median survival of 2–4 years and 5-year survival of less than 25%; the toxicity of chemotherapy is well known by clinicians and patients alike. Furthermore, our findings reflect previous qualitative research describing the focus in patients with mBC shifting from disease eradication to prolonging survival, alleviating symptoms, relying on mindfulness and spirituality, contemplating the future, and improving quality of life by differentiating surviving from living (14, 15).

As oncology research progressively looks to target the process of metastasis, which is responsible for the majority of treatment failures, new targeted therapies will gradually emerge and be incorporated into clinical practice, because the existence of a target facilitates the process of approval and reimbursement of new drugs. However, innovative technologies, such as social media listening, generate huge data sets that are not currently used in regulatory procedures and drug toxicity monitoring. These data have the potential to provide a patient voice in the regulation of new and existing medicines.

### Strengths and Limitations

All social media posts that met the predefined search strategy were subjected to automated checks, with an additional layer of human intelligence to screen for irrelevant material prior to content analysis. This methodology permitted large amounts of data to be analyzed quickly and efficiently, allowing contextualized data to be categorized into insightful and non-insightful data. Previously cited limitations of social media listening studies have included the inability to capture human expression (23). Talkwalker natural language processing software allowed a sentiment analysis to be performed, determining the deeper context of social media discussions and categorizing them into positive, negative, or neutral connotations. The social media listening approach used in this study, therefore, allowed a large body of user-generated content across 14 European countries to be analyzed. It allowed for the lived experience to be explored through open-ended information gathering.

Retrospective data collection inherently limits the amount of demographic or clinical information that can be obtained from the population sample. For example, the stage of disease was not well characterized, making it difficult to interpret results with respect to treatment patterns, sequencing, and effectiveness from the patient perspective. Furthermore, the retrospective analysis does not allow for specific issues, such as treatment tolerance and patient–clinician decision-making, to be actively explored or followed up through probing questions, nor could certain valuable data points, such as age, be captured. In this study, retrospective analysis of data from closed forums, such as breast cancer-specific Facebook groups, was unattainable, meaning a potentially large data set of mBC-related conversations could add to the authors’ findings.

It is recognized that mBC is more commonly diagnosed in patients 50–69 years of age (median 62 years at diagnosis) (32); however, the incidence trend of mBC in women younger than 40 years is increasing (33). The highest number of conversations was identified in social media users 31–40 years of age (*n* = 86; 37%), which is in line with social media use statistics. It could be speculated that the views and experiences of this population may not be indicative of the wider community. However, 59% of conversations in which age was identifiable were authored by stakeholders older than 41 years. This correlates with data published by Sadah et al., who reported that users of health-related social media platforms, including health forums and drug review websites, are generally older than users of general-purpose social forums (34). The experiences of stakeholders with mBC are probably proportionately represented in this social media listening study.

## Conclusion

Improvements in the detection and treatment of breast cancer have had a positive impact on overall survival rates in men and women with the disease. However, mBC continues to have a poor prognosis, which can have a significant impact on health-related quality of life. Social media represents a new dimension to healthcare, allowing rapid communication and sharing of literature data both between clinicians and patients and between patients themselves.

As a research tool, social media listening allows for the lived experience to be explored through open-ended information gathering. In this study, a lack of effective treatments for mBC, maintaining the quality of life, the social impacts of breast cancer, and the debilitating side effects of treatment all emerged as key themes, providing perspectives and insights into living with or caring for someone with mBC. Furthermore, volume trend analysis demonstrated an increased volume of mBC-related conversations during notable holidays or health campaigns. Collectively, these findings complement the qualitative literature on the lived experience of breast cancer.

During our analysis, the impact of the dramatic shift in healthcare priorities towards COVID-19 on mBC stakeholders and the effect on healthcare policies and practices across Europe was evident. Stakeholders expressed frustration about treatment delays and discontinuation. These findings correlated with Jerusalem et al., whereby cancellation or delay of surgery and chemotherapy were reported as the most affected treatment modalities of the COVID-19 pandemic.

Social media listening studies are therefore important for the training of healthcare professionals in communication and the importance of quality of life, the organization of healthcare and policy, and even for the design of clinical trials. However, the huge data sets generated by innovative technologies are not currently used in regulatory procedures and drug toxicity monitoring. If we are to optimally use the rich data collected on the lived experience of mBC, social media listening studies need to be utilized to support the regulation of new and existing medicines.

## Data Availability Statement

The original contributions presented in the study are included in the article/**Supplementary Material**. Further inquiries can be directed to the corresponding author.

## Author Contributions

All authors contributed equally to this work and agree to be accountable for the content of the work.

## Funding

This study and editorial assistance, provided by Oxford PharmaGenesis, Oxford, UK, were supported by Novartis Pharma AG, Basel, Switzerland. The funder was not involved in the study design, collection, analysis, interpretation of data, the writing of this article or the decision to submit it for publication.

## Conflict of Interest

MM has provided consultancy/advice or received reimbursement for travel expenses from Roche, Pfizer, Novartis, Lilly, and Gentili. MP confirms the absence of commercial and financial relationships. SA and JC are employees of Novartis Pharma AG. AS is an employee and stakeholder of Novartis Pharma AG. CV has provided consultancy/advice or received reimbursement for travel expenses from Grunenthal, Genentech/Roche, Pfizer, Novartis, Lilly, BMS, MSD, and Merck Serono.

## Publisher’s Note

All claims expressed in this article are solely those of the authors and do not necessarily represent those of their affiliated organizations, or those of the publisher, the editors and the reviewers. Any product that may be evaluated in this article, or claim that may be made by its manufacturer, is not guaranteed or endorsed by the publisher.

## Supplementary Material

Appendix 1 Search strings.

## References

[1] Jacot W Heudel PE Fraisse J Gourgou S Guiu S Dalenc F . Real-Life Activity of Eribulin Mesylate Among Metastatic Breast Cancer Patients in the Multicenter National Observational Esme Program. Int J Cancer (2019) 145(12):3359–69. doi: 10.1002/ijc.32402 31087564

[2] Simon J Chaix M Billa O Kamga AM Roignot P Ladoire S . Survival in Patients With Hr+/Her2- Metastatic Breast Cancer Treated With Initial Endocrine Therapy Versus Initial Chemotherapy. A French Population-Based Study. Br J Cancer (2020) 123(7):1071–7. doi: 10.1038/s41416-020-0979-3 PMC7525445 32678278

[3] Davie A Carter GC Rider A Pike J Lewis K Bailey A . Real-World Patient-Reported Outcomes of Women Receiving Initial Endocrine-Based Therapy for Hr+/Her2- Advanced Breast Cancer in Five European Countries. BMC Cancer (2020) 20(1):855. doi: 10.1186/s12885-020-07294-2 32894087 PMC7487722

[4] Gamucci T Mentuccia L Natoli C Sperduti I Cassano A Michelotti A . A Real-World Multicentre Retrospective Study of Paclitaxel-Bevacizumab and Maintenance Therapy as First-Line for Her2-Negative Metastatic Breast Cancer. J Cell Physiol (2017) 232(6):1571–8. doi: 10.1002/jcp.25685 PMC6220933 27861874

[5] Palleschi M Maltoni R Ravaioli S Vagheggini A Mannozzi F Fanini F . Ki67 and Pr in Patients Treated With Cdk4/6 Inhibitors: A Real-World Experience. Diagn (Basel) (2020) 10(8):573. doi: 10.3390/diagnostics10080573 PMC7460222 32784518

[6] Hartkopf AD Huober J Volz B Nabieva N Taran FA Schwitulla J . Treatment Landscape of Advanced Breast Cancer Patients With Hormone Receptor Positive Her2 Negative Tumors - Data From the German Praegnant Breast Cancer Registry. Breast (2018) 37:42–51. doi: 10.1016/j.breast.2017.10.002 29100043

[7] Adamo V Ricciardi GRR Giuffrida D Scandurra G Russo A Blasi L . Eribulin Mesylate Use as Third-Line Therapy in Patients With Metastatic Breast Cancer (Vespry): A Prospective, Multicentre, Observational Study. Ther Adv Med Oncol (2019) 11:1758835919895755. doi: 10.1177/1758835919895755 31903098 PMC6923689

[8] Garcia-Trevijano Cabetas M Lucena Martinez P Jimenez Nacher I Diaz Almiron M Zamora Aunon P Herrero Ambrosio A . Real-World Experience of Palbociclib and Ribociclib: Novel Oral Therapy in Metastatic Breast Cancer. Int J Clin Pharm (2021) 43(4):893–9. doi: 10.1007/s11096-020-01193-z 33170404

[9] Jacquet E Lardy-Cleaud A Pistilli B Franck S Cottu P Delaloge S . Endocrine Therapy or Chemotherapy as First-Line Therapy in Hormone Receptor-Positive Her2-Negative Metastatic Breast Cancer Patients. Eur J Cancer (2018) 95:93–101. doi: 10.1016/j.ejca.2018.03.013 29655061

[10] Bighin C Dozin B Poggio F Ceppi M Bruzzi P D'Alonzo A . Hormonal Therapy Followed by Chemotherapy or the Reverse Sequence as First-Line Treatment of Hormone-Responsive, Human Epidermal Growth Factor Receptor-2 Negative Metastatic Breast Cancer Patients: Results of an Observational Study. Oncotarget (2017) 8(27):44800–10. doi: 10.18632/oncotarget.14735 PMC5546519 28108743

[11] Moser A Korstjens I . Series: Practical Guidance to Qualitative Research. Part 3: Sampling, Data Collection and Analysis. Eur J Gen Pract (2018) 24(1):9–18. doi: 10.1080/13814788.2017.1375091 29199486 PMC5774281

[12] McKenna SP . Measuring Patient-Reported Outcomes: Moving Beyond Misplaced Common Sense to Hard Science. BMC Med (2011) 9:86. doi: 10.1186/1741-7015-9-86 21756344 PMC3170214

[13] Gill P Stewart K Treasure E Chadwick B . Methods of Data Collection in Qualitative Research: Interviews and Focus Groups. Br Dent J (2008) 204(6):291–5. doi: 10.1038/bdj.2008.192 18356873

[14] Mortensen G Madsen IB Krogsgaard R Ejlertsen B . Quality of Life and Care Needs in Women With Estrogen Positive Metastatic Breast Cancer: A Qualitative Study. Acta Oncol (2018) 57(1):146–51. doi: 10.1080/0284186X.2017.1406141 29202668

[15] Krigel S Myers J Befort C Krebill H Klemp J . 'Cancer Changes Everything!' Exploring the Lived Experiences of Women With Metastatic Breast Cancer. Int J Palliat Nurs (2014) 20(7):334–42. doi: 10.12968/ijpn.2014.20.7.334 25062379

[16] Kapoor K Tamilmani K Rana N Patil P Dwivedi Y Nerur S . Advances in Social Media Research: Past, Present and Future. Inf Syst Front (2017) 20:531–58. doi: 10.1007/s10796-017-9810-y

[17] Perrin A . Social Media Usage: 2005-2015. Pew Research Center (2015). Available at: http://www.pewinternet.org/2015/10/08/2015/Social-Networking-Usage-2005-2015/.

[18] Hunsaker A Hargittai E . A Review of Internet Use Among Older Adults. New Media Soc (2018) 20(10):3937–54. doi: 10.1177/1461444818787348

[19] Gualtieri L Akhtar F eds. (2013). Cancer Patient Blogs: How Patients, Clinicians, and Researchers Learn From Rich Narratives of Illness, in: Proceedings of the ITI 2013 35th International Conference on Information Technology Interfaces. Cavtat (Croatia): IEEE.

[20] Tsuya A Sugawara Y Tanaka A Narimatsu H . Do Cancer Patients Tweet? Examining the Twitter Use of Cancer Patients in Japan. J Med Internet Res (2014) 16(5):e137. doi: 10.2196/jmir.3298 24867458 PMC4060148

[21] Metwally O Blumberg S Ladabaum U Sinha SR . Using Social Media to Characterize Public Sentiment Toward Medical Interventions Commonly Used for Cancer Screening: An Observational Study. J Med Internet Res (2017) 19(6):e200. doi: 10.2196/jmir.7485 28592395 PMC5480009

[22] Cook NS Kostikas K Gruenberger JB Shah B Pathak P Kaur VP . Patients' Perspectives on Copd: Findings From a Social Media Listening Study. ERJ Open Res (2019) 5(1). doi: 10.1183/23120541.00128-2018 PMC6368996 30775374

[23] Wolffsohn JS Leteneux-Pantais C Chiva-Razavi S Bentley S Johnson C Findley A . Social Media Listening to Understand the Lived Experience of Presbyopia: Systematic Search and Content Analysis Study. J Med Internet Res (2020) 22(9):e18306. doi: 10.2196/18306 32955443 PMC7536603

[24] Shimkhada R Attai D Scheitler AJ Babey S Glenn B Ponce N . Using a Twitter Chat to Rapidly Identify Barriers and Policy Solutions for Metastatic Breast Cancer Care: Qualitative Study. JMIR Public Health Surveill (2021) 7(1):e23178. doi: 10.2196/23178 33315017 PMC7872835

[25] Hamm MP Chisholm A Shulhan J Milne A Scott SD Given LM . Social Media Use Among Patients and Caregivers: A Scoping Review. BMJ Open (2013) 3(5). doi: 10.1136/bmjopen-2013-002819 PMC3651969 23667163

[26] Humphrey L Willgoss T Trigg A Meysner S Kane M Dickinson S . A Comparison of Three Methods to Generate a Conceptual Understanding of a Disease Based on the Patients' Perspective. J Patient Rep Outcomes (2017) 1(1):9. doi: 10.1186/s41687-017-0013-6 29757313 PMC5934934

[27] Kaleem T Malouff TD Stross WC Waddle MR Miller DH Seymour AL . Google Search Trends in Oncology and the Impact of Celebrity Cancer Awareness. Cureus (2019) 11(8):e5360. doi: 10.7759/cureus.5360 31608195 PMC6783227

[28] Evans DG Barwell J Eccles DM Collins A Izatt L Jacobs C . The Angelina Jolie Effect: How High Celebrity Profile Can Have a Major Impact on Provision of Cancer Related Services. Breast Cancer Res (2014) 16(5):442. doi: 10.1186/s13058-014-0442-6 25510853 PMC4303122

[29] Liede A Cai M Crouter TF Niepel D Callaghan F Evans DG . Risk-Reducing Mastectomy Rates in the Us: A Closer Examination of the Angelina Jolie Effect. Breast Cancer Res Treat (2018) 171(2):435–42. doi: 10.1007/s10549-018-4824-9 PMC6096880 29808287

[30] Basu NN Hodson J Chatterjee S Gandhi A Wisely J Harvey J . The Angelina Jolie Effect: Contralateral Risk-Reducing Mastectomy Trends in Patients at Increased Risk of Breast Cancer. Sci Rep (2021) 11(1):2847. doi: 10.1038/s41598-021-82654-x 33531640 PMC7854742

[31] Onesti CE Tagliamento M Curigliano G Harbeck N Bartsch R Wildiers H . Expected Medium- and Long-Term Impact of the Covid-19 Outbreak in Oncology. JCO Glob Oncol (2021) 7:162–72. doi: 10.1200/GO.20.00589 PMC8081548 33529077

[32] Chen MT Sun HF Zhao Y Fu WY Yang LP Gao SP . Comparison of Patterns and Prognosis Among Distant Metastatic Breast Cancer Patients by Age Groups: A Seer Population-Based Analysis. Sci Rep (2017) 7(1):9254. doi: 10.1038/s41598-017-10166-8 28835702 PMC5569011

[33] Johnson RH Chien FL Bleyer A . Incidence of Breast Cancer With Distant Involvement Among Women in the United States, 1976 to 2009. JAMA (2013) 309(8):800–5. doi: 10.1001/jama.2013.776 23443443

[34] Sadah SA Shahbazi M Wiley MT Hristidis V . A Study of the Demographics of Web-Based Health-Related Social Media Users. J Med Internet Res (2015) 17(8):e194. doi: 10.2196/jmir.4308 26250986 PMC4705027

